# A validated cellular biobank for β-thalassemia

**DOI:** 10.1186/s12967-016-1016-4

**Published:** 2016-09-02

**Authors:** Lucia Carmela Cosenza, Laura Breda, Giulia Breveglieri, Cristina Zuccato, Alessia Finotti, Ilaria Lampronti, Monica Borgatti, Francesco Chiavilli, Maria Rita Gamberini, Stefania Satta, Laura Manunza, Franca Rosa De Martis, Paolo Moi, Stefano Rivella, Roberto Gambari, Nicoletta Bianchi

**Affiliations:** 1 Department of Life Sciences and Biotechnology, Section of Biochemistry and Molecular Biology, University of Ferrara, Via Fossato di Mortara 74, 44121 Ferrara, Italy; 2 Department of Hematology-Oncology, Weill Cornell Medical College, New York, NY USA; 3 Department of Hematology, Children’s Hospital of Philadelphia, 3615 Civic Center Blvd, Abramson Research Center Philadelphia, Philadelphia, PA 19104 USA; 4 Laboratory for the Development of Gene and Pharmacogenomic Therapy of Thalassemia, Biotechnology Centre of Ferrara University, Ferrara, Italy; 5 Servizio di Immunoematologia e Trasfusione, S.I.T., ULSS 18, Rovigo, Italy; 6 Dipartimento di Scienze Mediche-Pediatria, Università di Ferrara, Ferrara, Italy; 7 Clinica Pediatrica 2a, Dipartimento di Sanità Pubblica, Medicina Clinica e Molecolare, Università di Cagliari, Ospedale Regionale Microcitemie ASL8, Cagliari, Italy

**Keywords:** Thalassemia, Biobanking, HbF induction, Gene therapy

## Abstract

**Background:**

Cellular biobanking is a key resource for collaborative networks planning to use same cells in studies aimed at solving a variety of biological and biomedical issues. This approach is of great importance in studies on β-thalassemia, since the recruitment of patients and collection of specimens can represent a crucial and often limiting factor in the experimental planning.

**Methods:**

Erythroid precursor cells were obtained from 72 patients, mostly β-thalassemic, expanded and cryopreserved. Expression of globin genes was analyzed by real time RT-qPCR. Hemoglobin production was studied by HPLC.

**Results:**

In this paper we describe the production and validation of a Thal-Biobank constituted by expanded erythroid precursor cells from β-thalassemia patients. The biobanked samples were validated for maintenance of their phenotype after (a) cell isolation from same patients during independent phlebotomies, (b) freezing step in different biobanked cryovials, (c) thawing step and analysis at different time points. Reproducibility was confirmed by shipping the frozen biobanked cells to different laboratories, where the cells were thawed, cultured and analyzed using the same standardized procedures. The biobanked cells were stratified on the basis of their baseline level of fetal hemoglobin production and exposed to fetal hemoglobin inducers.

**Conclusion:**

The use of biobanked cells allows stratification of the patients with respect to fetal hemoglobin production and can be used for determining the response to the fetal hemoglobin inducer hydroxyurea and to gene therapy protocols with reproducible results.

**Electronic supplementary material:**

The online version of this article (doi:10.1186/s12967-016-1016-4) contains supplementary material, which is available to authorized users.

## Background

Biobanking of biological materials, including viable cells, is a new and very relevant approach which involves a wide range of public and private institutions [1]. The number of biobanks is rapidly increasing worldwide, helping to create collaborative networks. The creation of these banks can tackle very important biomedical issues that require large numbers of tissue samples of the same groups of pathologies. In Europe, more than 225 biobanks and institutions from over 30 countries that collect samples and pathological/clinical data belong to the Biobanking and Biomolecular Resources Research Infrastructure (BBMRI) [1]. Biobanking of biological material (cellular pellets, DNA, RNA) is important to organize collection of high-quality samples with reliable clinical information for diagnostics, therapy and research. On the other hand, highly informative experiments with biological modifiers could be performed, at least in theory, only using validated biological material collected within cellular biobank [2, 3].

Cellular biobanking is a key step in a variety of “OMICS” analyses, as well as in the screening of bioactive molecules for the development of novel therapeutic protocols [4]. This is particularly important to test a variety of treatments on primary cells isolated from affected patients in order to develop novel diagnostic, prognostic and therapeutic approaches, toward personalized treatments. The biological assay used to test therapeutic molecules on primary erythroid cells from β-thalassemia patients is based on one method called “two-phase culture procedure” developed by the Fibach’s research group [5, 6]. In this system, peripheral blood mononuclear cells (PBMCs) isolated from 20 to 25 ml of patients’ blood are seeded in a Phase I culture for about 7 days and then differentiated into hemoglobin-producing erythroid cell for a further 7–10 day period (Phase II) by exposure to erythropoietin [5, 6]. This protocol has been widely applied to develop novel fetal hemoglobin (HbF) inducers [7, 8], to validate gene therapy strategies based on novel lentiviral vectors [9, 10] and to perform gene editing using approaches based on ZFN, TALEN, and CRISPR-CAS9 [11–15].

A limitation of the two-phase culture and its use for biomedical approaches is the restricted yield of erythroid precursors cells (ErPCs) that can be obtained, because the two-phase culture cannot be proposed for parallel culturing of high number of ErPCs, in consideration of the need of blood sampling from patients, who undergo blood transfusion with different time frequency and scheduled time periods. With these considerations in mind, the generation of a cellular biobank that includes cellular expansion, freezing, cryopreservation, storage and, finally, subculturing after thawing, represents an innovative methodology to perform experiment in parallel and without time constraint.

In this manuscript we describe (a) the generation of a cellular biobank (Thal-Biobank) from β-thalassemia patients; (b) its characterization with respect to maintenance of the phenotype (% of HbF production); (c) its validation, using frozen cryopreserved samples for thawing and subculturing in different laboratories and by testing induction of HbF using hydroxyurea (HU). HU was chosen among the different available HbF inducers, since (a) it is already used in experimental therapy of patients affected by β-thalassemia and sickle-cell anemia and (b) it is the only drug approved by US Food and Drug Administration for Sickle-cell disease (SCD) patients [16, 17].

## Methods

### Patients

Patients were recruited and blood samples obtained according to the Declaration of Helsinki and following specific approvals of the study by the Ethical Committees of Ferrara Hospital and Rovigo Hospital. The blood samples were collected from β-thalassemia patients after signature of the informed consent form.

### Isolation and culture of peripheral blood cells (Protocol A)

Peripheral blood mononuclear cells (PBMCs) (from about 25 ml of blood) were collected in Vacutainer LH treated tubes (BD Vacutainers, Becton–Dickinson, UK). PBMCs isolation was obtained from whole blood by Ficoll-Hypaque density gradient centrifugation (Lympholyte®-H Cell Separation Media, Cedarlane, Euroclone, Italy). After the separation of the various blood components, the ring was harvested and washed once with 1× Dulbecco’s Phosphate Buffered Saline without Ca & Mg (DPBS W/O CA-MG, GIBCO, Invitrogen, Life Technologies, Carlsbad, CA, USA). CD34^+^ cells were selected from PBMCs using anti-CD34^+^ magnetic microbeads and magnetic activated cells sorting separation LS columns (both from Miltenyi Biotec, Bergisch Gladbach, Germany) according to the manufacturer’s protocol. The separation in the column was done twice to increase the CD34^+^ cells purification. The obtained cells were maintained in culture with two different protocols. In the first protocol (Protocol A), the medium contained α-minimal essential medium (α-MEM, Sigma-Genosys, Saint Louis, Missouri, USA), prepared from a powder and diluted with water; a solution of Penicillin-Streptomycin (PEN-STREP 10,000 U/mL, Lonza, Verviers, Belgium); 10 % fetal bovine serum (FBS, Celbio, Milan, Italy); 10 % conditioned medium (CM), obtained from cell cultures of bladder cancer cells (5637); 1 µg/ml of cyclosporin A (Sigma-Aldrich, Saint Louis, Missouri, USA), prepared from cyclosporine absolute ethanol and diluted in 1X DPBS (GIBCO, Invitrogen, Life Technologies Carlsbad, CA, USA), in the ratio 1:1. After 7 days in Phase I culture, the non adherent cells were washed once with 1X DPBS (GIBCO, Invitrogen, Life Technologies Carlsbad, CA, USA), and then cultured in Phase II medium. This medium contains α-MEM (Sigma Genosys, Cambridge, UK), 30 % FBS (Celbio, Milan, Italy), 1 % deionized bovine serum albumin (BSA, Sigma Genosys, Cambridge, UK), 10^−5^ M β-mercaptoethanol (Sigma Genosys, Cambridge, UK), 2 mM l-glutamine (Sigma Genosys, Cambridge, UK), 10^−6^ M dexamethasone (Sigma Genosys, Cambridge, UK), and 1 U/mL human recombinant erythropoietin (EPO Tebu-bio, Magenta, Milan, Italy), and stem cell factor (SCF, BioSource International, Camarillo, CA, USA) at the final concentration of 10 ng/mL.

### Isolation and culture of peripheral blood cells (Protocol C)

In the second protocol, named Protocol C, the isolation of mononuclear cells was performed starting from 20–25 ml of peripheral blood collected before transfusion from patients who gave informed consent. A mixture of Blood and PBS 1× at a 1:1.5 ratio was stratified on top of Lympholyte®-H Cell Separation Media. A monolayer of PBMCs was obtained by centrifugation at 2000 rpm for 30 min, at room temperature, and carefully transferred into new tube. PBMCs were washed twice in 50 ml PBS 1×, and resuspended in 600 μl of BSA solution.

The isolation of CD34^+^ cells from PBMCs was performed using the CD34^+^ MicroBead Kit, LS MiniMACS Column, by magnetic separation using an autoMACS Separator (all from Miltenyi Biotec). 100 μl of CD34^+^ MicroBead were added to the cell suspension and gently mixed for 15 min at 4 °C. Two washes were performed before proceeding with column separation. Cells were resuspended in 1 mL of beading buffer (PBS1X, 2mMEDTA, 0.5 %BSA) and loaded onto the column in two consecutive steps with beading buffer washes in between. To enrich for CD34^+^ cells, the eluted fraction was re-eluted into a second LS Column. Cells pellets were resuspended in 5 ml of growth medium.

Five ml of expansion medium contains: StemSpan Serum-Free Medium Expansion (Voden, Vancouver, Canada), 50 µl of StemSpan® CC100 Cytokine Cocktail for Expansion of Human Hematopoietic Cells Stem Cell Technologies (Voden, Vancouver, Canada), 2 U/mL erythropoietin (EPO, Tebu-bio, Magenta, Milan, Italy), 10^−6^ M dexamethasone (Dexamethasone 21-phosphate disodium salt, Sigma-Aldrich, Saint Louis, Missouri, USA), 50 µl of a 100× Pennicilin/Streptomycin solution (Lonza, Verviers, Belgium). Cell growth and differentiation was monitored over time. Fresh expansion medium was added to maintain cell confluence below 5 × 10^5^ cells/mL. Cells were frozen between 7 and 12 days of expansion.

### Freezing, cryopreservation and thawing of peripheral blood cells isolated following Protocol C

Once the maximum in cell expansion was achieved, CD34^+^ cells were frozen in single vials of 5 × 10^6^ cells each, following a previously described method [18] using a solution made of: 40 % Iscove’s modified Dulbecco’s Medium (IMDM, Life Tecnologies, Carlsbad, CA, USA), 50 % FBS (Celbio, Milan, Italy) and 10 % Dimethyl Sulfoxide RPE-ACS (DMSO, Carlo Erba, Italy).

Cells were thawed by immediate incubation at 37 °C and resuspended dropwise in Iscove Modified Dulbecco’s Medium (IMDM) with 5 % FBS. After a 10 min incubation at room temperature, the cell suspension was centrifuged at 1200 rpm at room temperature for 5 min, the supernatant removed and the cells suspended in expansion medium.

### Characterization of CD34^+^ ErPC cultures by Fluorescence-activated cell sorting (FACS) analysis

Phenotypic characterization of CD34^+^cells before and after cryopreservation, thawing and culturing in the presence of SCF and EPO was performed using FACS analyses using 200,000 cells and antibodies directed against the following markers: CD34, CD44, Glycophorin A (GPA), CD117, CD29. The ErPCs expansion has been analyzed by FACS using CD34 marker expression Allophycocyanin (APC) cojugated and CD44 monoclonal antibody FITC-conjugated before thawing. Erythroid differentiation of the ErPC cultures was investigated studying GPA expression by FACS analysis using polyclonal anti-human Glycophorin A antibody/Rabbit/IgG conjugated (Thermo schientific, Pierce, Rockford, USA) and Rabbit IgG-heavy and light chain cross-adsorbed antibody R-Phycoerythrin (PE) conjugated (Bethyl, Temaricerche, Italy). The other employed antibodies were anti-CD117 monoclonal antibody PE-cojugated (Exalpha Bilogical Inc., USA); anti-CD29 monoclonal antibody FITC-conjugated (Thermo schientific, Pierce, Rockford, USA); anti-CD44 monoclonal antibody FITC-conjugated (Exalpha Bilogical Inc., USA).

For FACS analysis, one μl of anti-human GPA antibody was added to freshly isolated cells on ice in 100 μl 1× PBS and 0.1 % fetal calf serum (FCS) for 30 min. Then, when appropriate, rabbit IgG-heavy and light chain cross-adsorbed R-PE conjugate antibody (Bethyl, Temaricerche, Italy) was added (1:50) to PBS-washed cells and a further incubation on ice (1× PBS, 0.1 % FBS) was carried out for 30 min; 20 μl of CD117 monoclonal antibody PE-conjugated, or both 10 μl of CD29 monoclonal antibody FITC-conjugated and CD44 monoclonal antibody FITC-conjugated were added to freshly isolated cells on ice in 100 μl 1× PBS and 0.1 % FCS for 30 min. Finally, cells were washed in 1× PBS and analyzed using the BD FACScan system (Becton, Dickinson & Company, Italy).

### Benzidine staining

The benzidine assay was used to evaluate erythroid differentiation. Five microlitres of 0.1 mM benzidine, resuspended in 2.86 % glacial acetic acid and activated with 33 % H_2_O_2_, were added to the same volume of the cells. The percentage of resulting blue cells indicates the level of erythroid differentiation.

### Treatment with hydroxyurea

About 4 days after thawing the CD34^+^ cells have been treated with hydroxyurea using a concentration previously tested on erythroleukemic K562 cells. We used 2–3 × 10^6^ cells for RNA extraction to T^0^. Then, the cells were washed twice with 1× DPBS (GIBCO, Invitrogen, Life Technologies Carlsbad, CA, USA). From the pellet obtained we have done hemoglobins analysis by HPLC (high performance liquid chromatography) and transcript analysis by RNA extraction.

### RT-PCR and qPCR analysis

The total cellular RNA was extracted by TRI Reagent® (Sigma-Aldrich, Saint Louis, Missouri, USA). The isolated RNA was washed once with cold 75 % ethanol, dried and dissolved in diethylpyrocarbonate-treated Water Molecular Biology Reagent (WMBR, nuclease free, Sigma-Aldrich, Saint Louis, Missouri, USA) before use. For gene expression analysis 1 μg of total RNA was reverse transcribed by using the TaqMan® Reverse Transcription Reagents and random hexamers (Applied Biosystems, Life Technologies, Carlsbad, CA, USA). Quantitative real time PCR assay, to quantify the expression of the globin genes, was carried out using gene-specific double-quenched probes. Probes are labeled in 5′ with different fluorochromes, whereas in 3′ the BHQ (Black Hole Quencher™) is present. Reaction mixture contained 1× iQTM Multiplex Powermix (Bio-Rad, Hercules, California, USA), 300 nM forward and reverse primers and the 200 nM probe. The assays were carried out in iCycler IQ5 (Bio-Rad, Hercules, CA, USA). After an initial denaturation at 95 °C for 30 s, the reactions were performed for 50 cycles (95 °C for 10 s, 60 °C for 45 s). To compare gene expression of each template amplified was used ΔΔCt method employing software IQ5 (Bio-Rad, Hercules, California, USA).

### High performance liquid chromatography (HPLC)

ErPCs were harvested, washed once with PBS and the pellets were lysed. After incubation on ice for 15 min, and spinning for 5 min at 14,000 rpm in a microcentrifuge, the supernatant was collected and injected. Hb proteins present in the lysates were separated by cation-exchange HPLC [19], using a Beckman Coulter instrument System Gold 126 Solvent Module-166 Detector. Hemoglobins were separated using a PolyLC (Columbia, MD, USA) PolyCAT-A model (35 mm × 4.6 mm) column; samples were eluted in a solvent gradient using aqueous sodium chloride-BisTris-KCN buffers and detection was performed at 415 nm. The standard controls were the purified HbA (SIGMA, St Louis, MO, USA) and HbF (Alpha Wassermann, Milano, Italy).

### Genomic DNA extraction

The DNA was extracted from 200–300 μL of whole blood using QiAmp DNA Mini Kit & QiAmp DNA Blood Mini Kit (QIAGEN, Hilden, Germany) according to manufacture’s protocol. The DNA obtained was visualized on a UV transilluminator after 0.8 % agarose gel electrophoresis and quantified using the spectrophotometer SmartSpec ™ Plus (Biorad Smartspec Plus, Bio-Rad, Hercules, California, USA).

### Polymerase chain reaction (PCR) and sequencing reaction

Β-globin gene was amplified starting from 300 ng of genomic DNA. Each reaction was carried out in a final volume of 100 μL, in the presence of 1× buffer (10 mM Tris–HCl pH 8.8, 1.5 mM MgCl2, 50 mM KCl, 0.1 % Triton X-100), 33 μM dNTPs, 0.25 μM forward and reverse primers, 2 U of DyNAzyme™ II DNA Polymerase (Finnzymes, Espoo, Finland) or DNA polymerase DreamTaq™ 5 U/µl (MBI Fermentas, Burlington, ON, Canada) and ultra-pure water. Each reaction was subjected to an initial denaturation step of 2 min at 94 °C. The 35 PCR cycles used were as follows: denaturation, 30 s at 94 °C; annealing, 30 s at 65 °C; elongation, 1 min at 72 °C. PCR products were analyzed by agarose-gel electrophoresis to 1 % before being purified for sequencing. The PCR products were displayed on a UV transilluminator after 1 % agarose gel electrophoresis. The PCR products were purified with MicroClean (Microzones Limited, Haywards Heath, West Sussex, UK) and were sequenced in both directions using the PCR primers forward and reverse and the BigDye^®^ Terminator version 1.1 Cycle Sequencing Kit (Life Technologies, Carlsbad, CA, USA). The reaction products were purified from unincorporated ddNTPs by using a 96-well MultiScreen™ (Merck Millipore KGaA, Darmstadt, Germany) plate containing Sephadex™ G-50 Superfine (Amersham Biosciences, UK). Sequencing was performed by BMR Genomics (Padua, Italy), while the obtained sequence data were analyzed by the Sequence Scanner, version 1.0 (Applied Biosystems, Life Tecnologies, Carlsbad, CA, USA) software.

### Synthetic oligonucleotides

The oligonucleotides used as primers in PCR and sequencing reactions were synthesized by Sigma Genosys (Cambridge, UK) and reported in Table 1. The RT-qPCR (reverse transcription quantitative polymerase chain reaction) primers and probes were purchased from Applied Biosystems (Life Technologies, Carlsbad, CA, USA) and reported in Table 2. All the oligonucleotides were designed using the software Primer Express™, version 2.2 (Perkin-Elmer, Applied Biosystems, Life Technologies, Carlsbad, CA, USA).

**Table 1:** Primers used in the polymerase chain reactions and in the sequencing reactions for β-globin gene

Primer	Sequence (5′–3′)	Tm (°C)
Forward primer BGF	5′-GTGCCAGAAGAGCCAAGGACAGG-3′	72.1
Forward primer T12F	5′-AGACCTCACCCTGTGGAGCC-3′	67.9
Forward primer T3F	5′-ACAATCCAGCTACCATTCTGCTTT-3′	65.7
Forward primer BG6F	5′-CGCTTTCTTGCTGTCCAATTTC-3′	66.7
Forward primer BG5SF	5′-GCCTGGCTCACCTGGACA-3′	66.7
Reverse primer BG4	5′-TCAGGAGTGGACAGATCCCC-3′	66.5
Reverse primer T12R	5′-AGTTCTCAGGATCCACGTGCA-3′	67.1
Reverse primer BGR	5′-CACTGACCTCCCACATTCCCTTTT-3′	69.8
Reverse primer BGi2R	5-‘GTTGCCCAGGAGCTGTGG-3′	67.1

For each primer the nucleotide sequence and melting temperature (Tm) have been reported

**Table 2:** Primers and probes employed in the multiplex quantitative real-time PCR

Primer	Sequence
Forward primer α-globin	5′-CGACAAGACCAACGTCAAGG-3′
Reverse primer α-globin	5′-GGTCTTGGTGGTGGGGAAG-3′
α-globin probe	5′-HEX-ACATCCTCTCCAGGGCCTCCG-BHQ-3′
Forward primer β-globin	5′-GGGCACCTTTGCCACAC-3′
Reverse primer β-globin	5′-GGTGAATTCTTTGCCAAAGTGAT-3′
β-globin probe	5′-Texas Red-ACGTTGCCCAGGAGCCTGAAG-BHQ-3′
Forward primer γ-globin	5′-TGACAAGCTGCATGTGGATC-3′
Reverse primer γ-globin	5′-TTCTTTGCCGAAATGGATTGC-3′
γ-globin probe	5′-FAM-TCACCAGCACATTTCCCAGGAGC-BHQ-3′
Forward primer RPL13A	5′-GGCAATTTCTACAGAAACAAGTTG-3′
Reverse primer RPL13A	5′-GTTTTGTGGGGCAGCATACC-3′
RPL13A *probe*	5′-CY5-CGCACGGTCCGCCAGAAGAT-BHQ-3′

The 5′ and 3′ chromogenic molecules are underlined in the sequence

## Results

### Production of the cellular Thal-Biobank

**Fig. 1:**
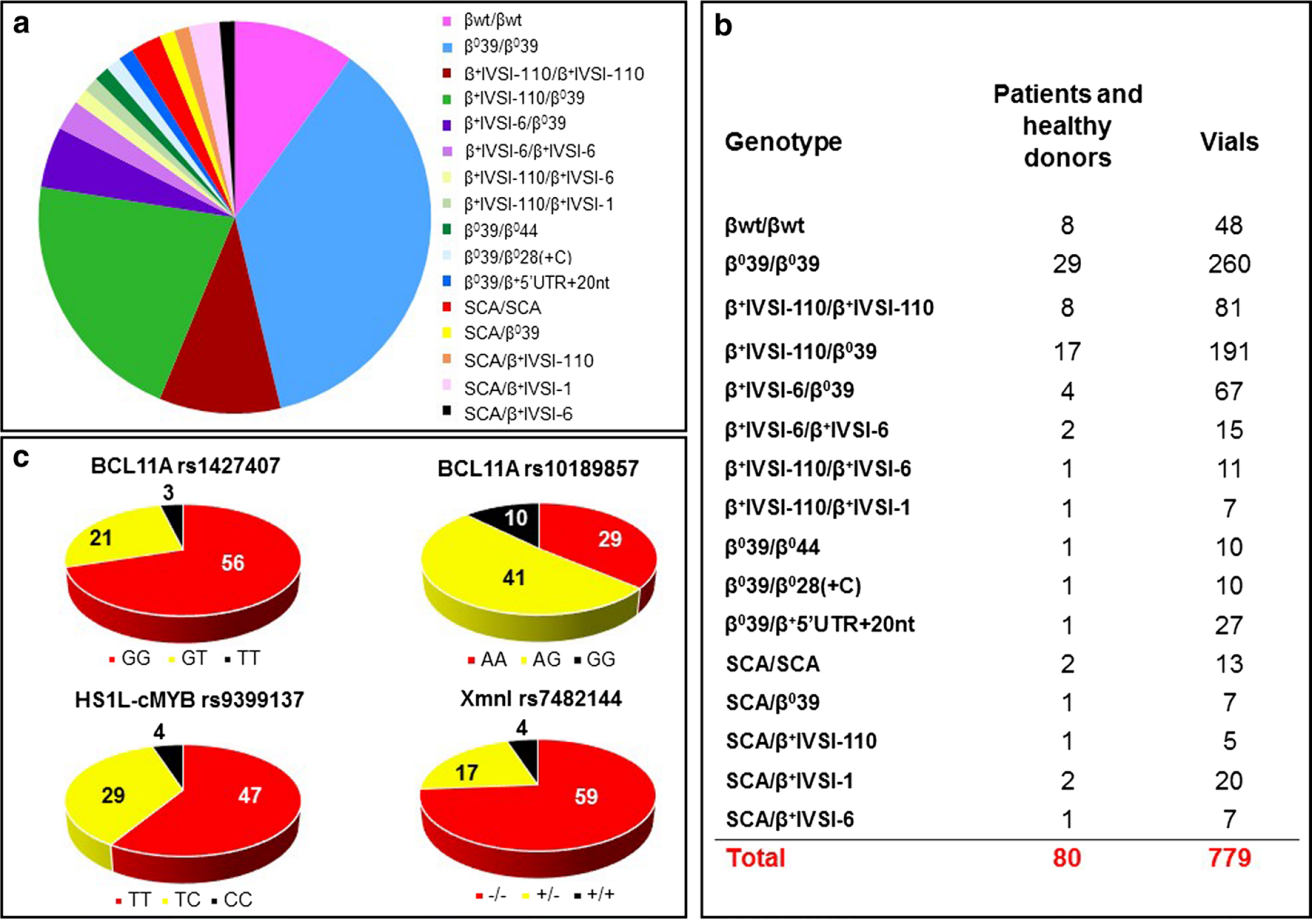
**a** Distribution of genotypes among consented patients affected by β-thalassemia and sickle-cell anemia (SCA) within the cellular Thal-Biobank. **b** Number of patients, related genotype and number of vials cryopreserved. **c** Distribution (expressed as percentage) of the *Xmn*I (*rs*7482144 −/+), BCL11A (*rs*1427407 G/T, *rs*10189857 A/G) and MYB (*rs9399137* C/T) polymorphisms

The cellular Thal-Biobank is composed of 779 biobanked specimens from 8 healthy donors and 72 patients with sickle cell anemia (SCA) and β-thalassemia. The complete list of biobanked cells and vials is reported in Additional file 1: Table S1, which includes for all the patients the genotype, the *Xmn*I (*rs*7482144 ±), two BCL11A (*rs*1427407 G/T, *rs*10189857 A/G) and one MYB (*rs9399137* C/T) polymorphisms. A summary of the composition of the cellular Thal-Biobank is reported in Fig. 1, which shows the distribution of the genotypes (1A and B) and related polymorphisms that are associated with modulation of HbF expression (1C). The most frequent genotypes are β^0^39/β^0^39 (29 patients), β^+^IVSI-110/β^0^39 (17 patients) and β^+^IVSI-110/β^+^IVSI-110 (8 patients).

### Kinetics of erythropoietin (EPO)-induced hemoglobin production following subculturing of cryopreserved ErPCs from β-thalassemia patients

**Fig. 2:**
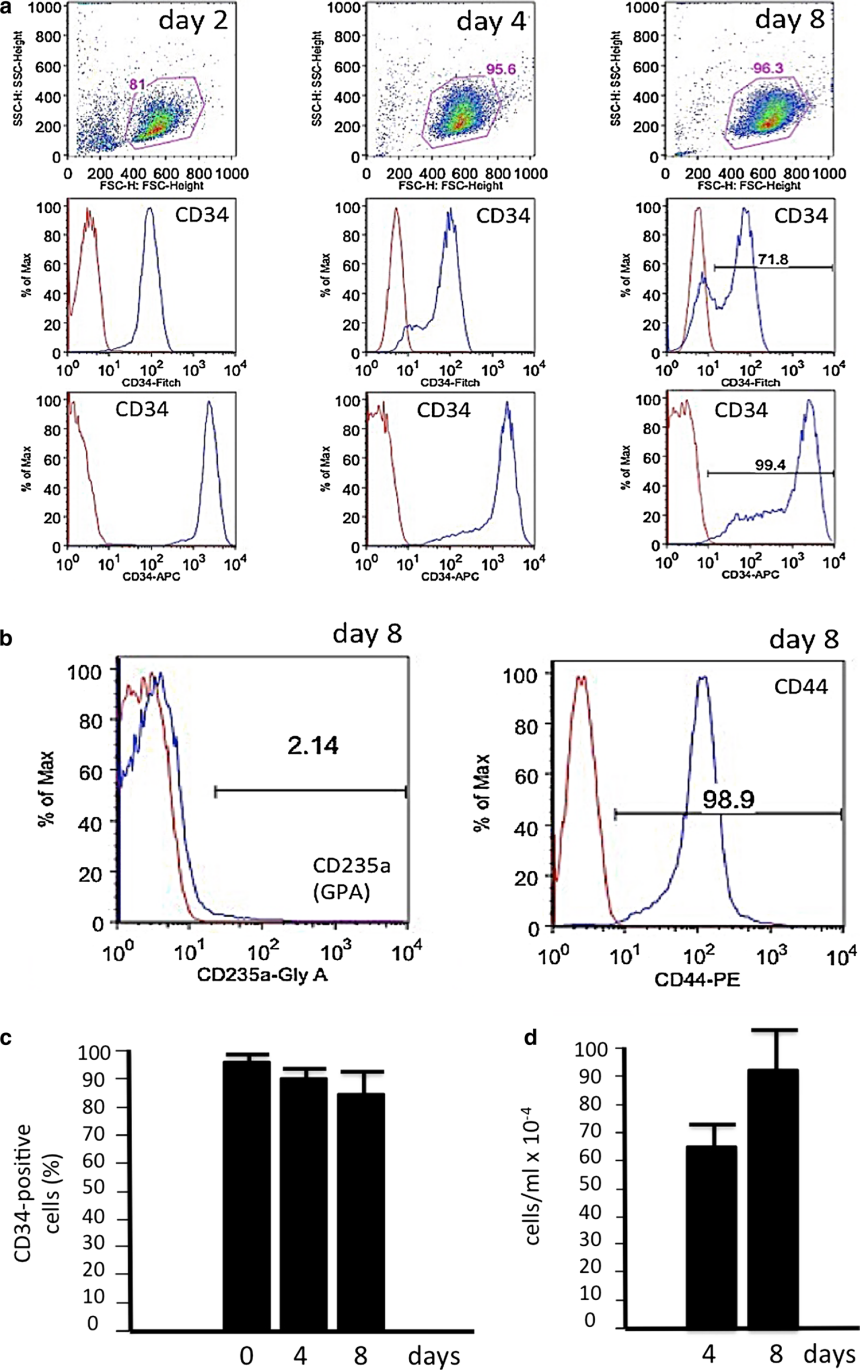
**a** Expression CD34 cell surface marker (FITC- or APC-conjugated antibody) in erythroid progenitor cells throughout expansion phase (Phase I) in Protocol C. **b** Expression of CD235a (GPA) and CD44 at day 8 of the expansion phase. Samples labeled with CD34-44-235a antibodies are represented in *blue* over IgG controls, in *red*. **c** Proportion of CD34-positive cells during the expansion time. Data represent the average ±SD from three independent expansion experiments. **d** Changes in cell number/ml from day 4 to day 8 after the thawing procedure. Data represent the average ±SD using four cryopreserved vials obtained from 4 different β-thalassemia patients

We cryopreserved only cells exceeding 90 % positivity for CD34 marker, starting from 7–8 days of expansion. Figure 2a shows a representative experiment indicating the proportion of CD34^+^ cells after 2, 4 and 8 days of phase I culture. As expected, at this stage cells do not express marker of erythroid differentiation (CD235a, or GPA) and still express high levels of CD44, an adhesion molecule that is reduced with erythroid progression (Fig. 2b). Quantitative data are shown in panel C of Fig. 2, while the cell growth potential from day 4 to day 8 of subculturing of the cryopreserved cells is shown in Fig. 2d.

**Fig. 3:**
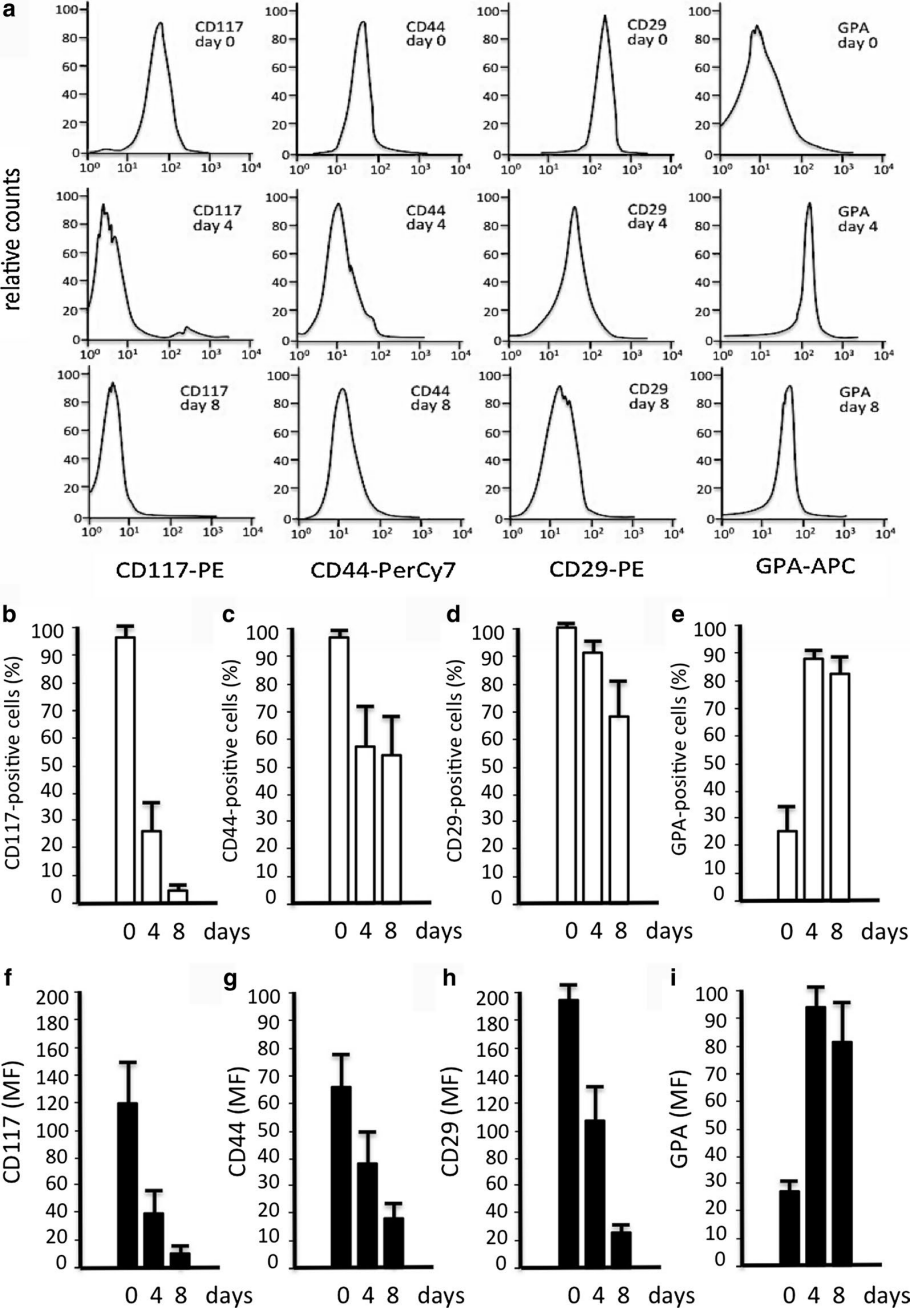
**a** Representative experiment showing the variation of expression of the hematopoietic stem cell marker CD117, adhesion molecule marker CD44, beta1-integrin surface marker CD29, and erythroid differentiation marker CD235 (GPA) in undifferentiated cells (day 0) and in cells at two progressive stages of erythroid maturation (day 4 and 8). **b**–**i** Quantitative data showing the % of cells expressing the indicated markers (**b**–**e**) and the mean fluorescence values (MF) (**f**–**i**) after FACS analyses using antibodies recognizing CD117 (**b**, **f**), CD44 (**c**, **g**), CD29 (**d**, **h**) and GPA (**e**, **i**). The data represent the average ±SD obtained in seven independent experiments using vials obtained from 7 different β-thalassemia patients

We characterized the cell phenotype over time by flow cytometry using a panel of antibodies recognizing “early” erythroid progenitor/adhesion markers (CD117, CD44, CD29) as well as a “late” erythroid marker (GPA) (Fig. 3). As erythroid maturation progresses a down regulation of CD117, CD44 and CD29 is observed (Fig. 3a–d, f–h) and concurrently an upregulation of GPA expression (Fig. 3a, e, i). Data obtained using CD71 (transferrin receptor 1) confirm that, as expected, this erythroid associated marker is present in nearly 100 % of the EPO-cultured ErPCs since day 4 of culture (not shown). Interestingly, the decrease of BFUe associated markers (i.e. CD44) and the increase of CFUe associated markers (i.e. GPA) are compatible with the BFUe → CFUe switch found in cultured erythroid progenitors by several research groups. This phenotypic characterization is very similar to that reported by Chen et al. [20], Li et al. [21] and Mori et al. [22].

**Fig. 4:**
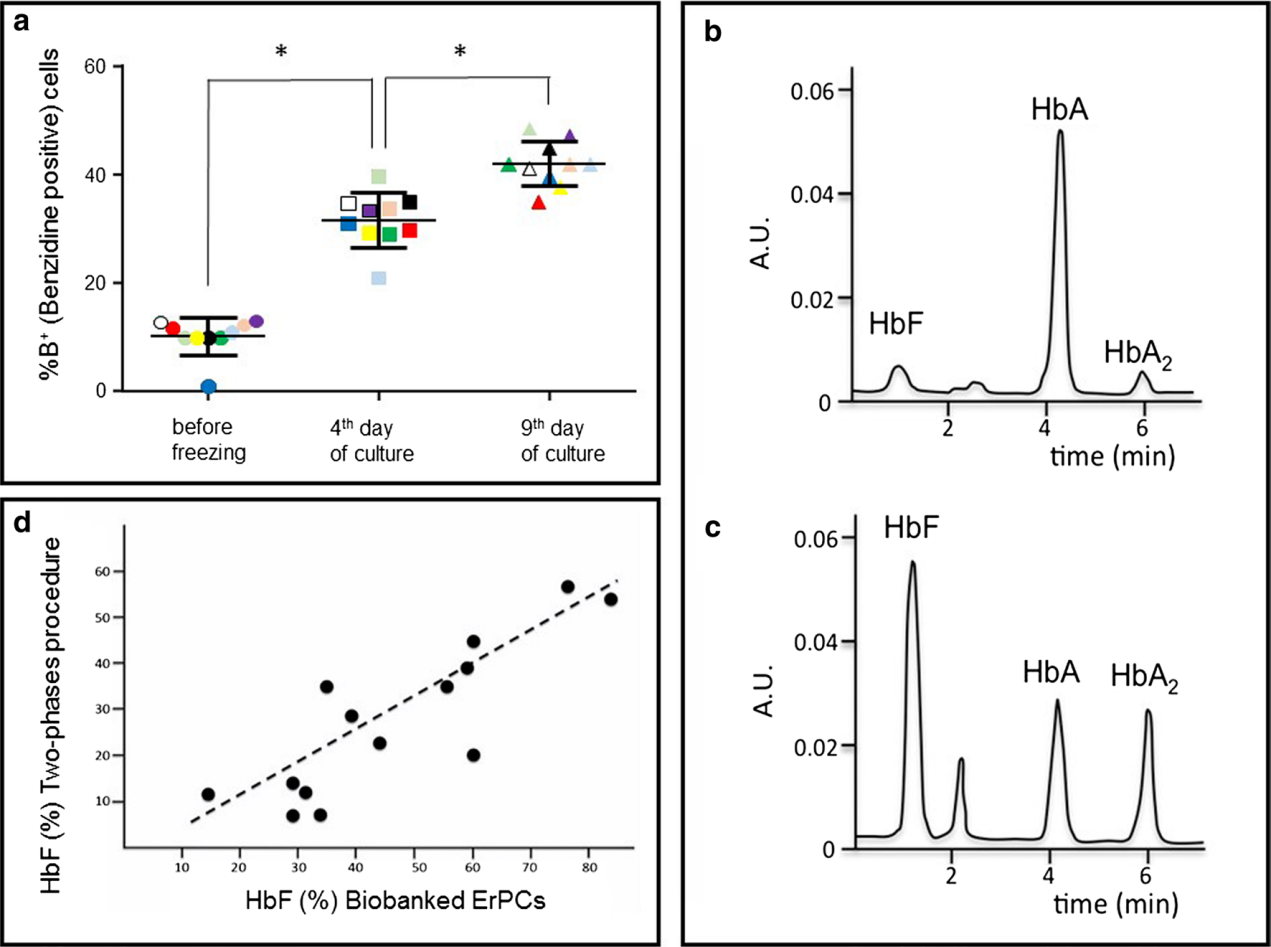
**a** Erythroid differentiation (% of benzidine-positive, haemoglobin containing cells) evaluated at different days (as indicated) of the cell cultures from different patients (*each color* represent one subject) (*P < 0.05). **b**, **c** HPLC analysis of the ErPCs cell cultures show representative examples of lysates containing moderate (**b**) and high (**c**) HbF endogenous levels. **d** Correlation between HbF levels determined using the two different cell culture protocols, the Fibach’s method (“Two-phases procedure”) and the protocol based on biobanked cells described in the present manuscript

Remarkably, the majority of the cryopreserved samples (more than 90 %) exhibit low proportion of benzidine-positive (hemoglobin containing) cells (Fig. 4a). In fact, the proportion of benzidine-positive cells at T_0_ was always less than 8–15 % in thawed samples. The progressive increase in the proportion of benzidine-positive cells at 4 and 9 days of subculturing in Phase II medium (Fig. 4a), confirms erythroid maturation measured by GPA staining.

Beside phenotypic characterizations of the cells (Figs. 2, 3, 4a), we were interested in determining whether this approach allows stratification of the patients with respect to HbF production. The level of HbF synthesis can greatly vary between patients’ ErPCs (Fig. 4b, c). When comparing baseline levels of HbF synthesis across samples, there is a good correlation between the HbF levels found in ErPCs induced following the classical two-phases differentiation protocols developed by the Fibach’s group and cryopreserved samples from same β-thalassemia patients, as shown in Fig. 4d.

### Biobanked cells originated from different blood sampling of a same β-thalassemia patient maintain the same hemoglobin pattern

**Fig. 5:**
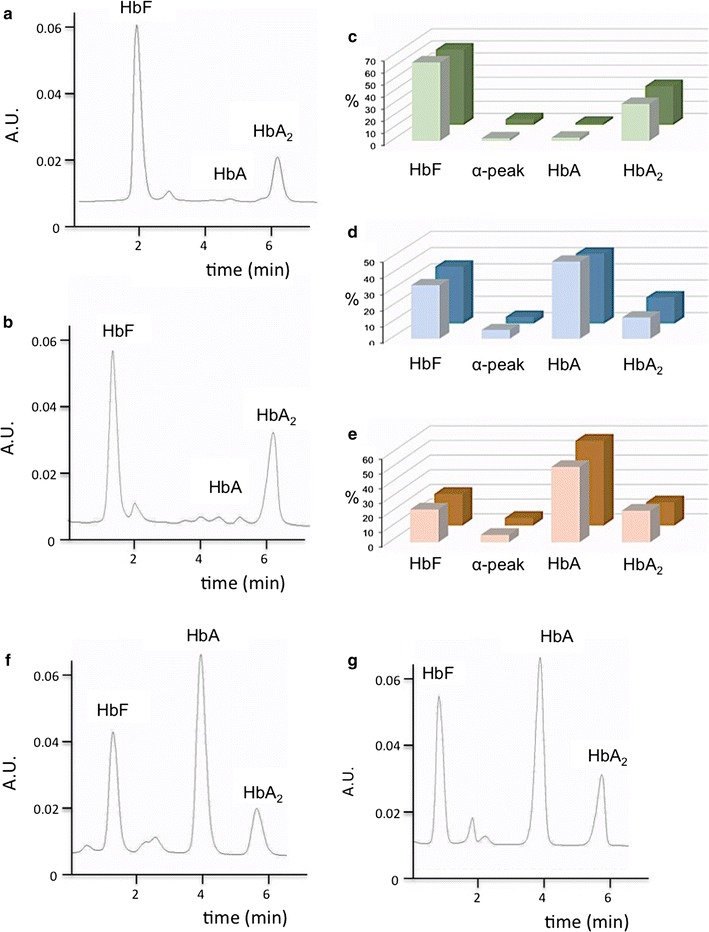
**a**, **b** Representative chromatograms obtained from HPLC analysis carried out on thawed cells collected from the same patient at two different times. **c**–**e** Hemoglobin profile in ErPCs collected at two time points from three patients. **c** AVLTF; **d** AVLTA and **e** AVLTQ. **f**, **g** HPLC profiles of representative lysates obtained from one sampling; in this case two batches of cells were thawed at different times

The first validation of the cellular Thal-Biobank was undertaken following careful analysis of the hemoglobin pattern produced by cryopreserved samples developed using blood sampling from a same patients performed at different periods of time. The HPLC analysis of the hemoglobin produced by cryopreserved and subcultured ErPCs isolated on March 2013 and November 2013 from a same patient shows that the hemoglobin pattern is stable (Fig. 5a, b). HPLC analyses on hemolysates from three different patients’s ErPCs corroborate this finding. Importantly, ErPCs derived from blood drawn from the same patient at different times maintain an almost identical pattern of hemoglobin production, indicating that this approach allows reproducible yield in Hb synthesis (Fig. 5c–e).

### Biobanked samples of a same β-thalassemia patient frozen and subcultured at different time periods maintain the same hemoglobin pattern

**Fig. 6:**
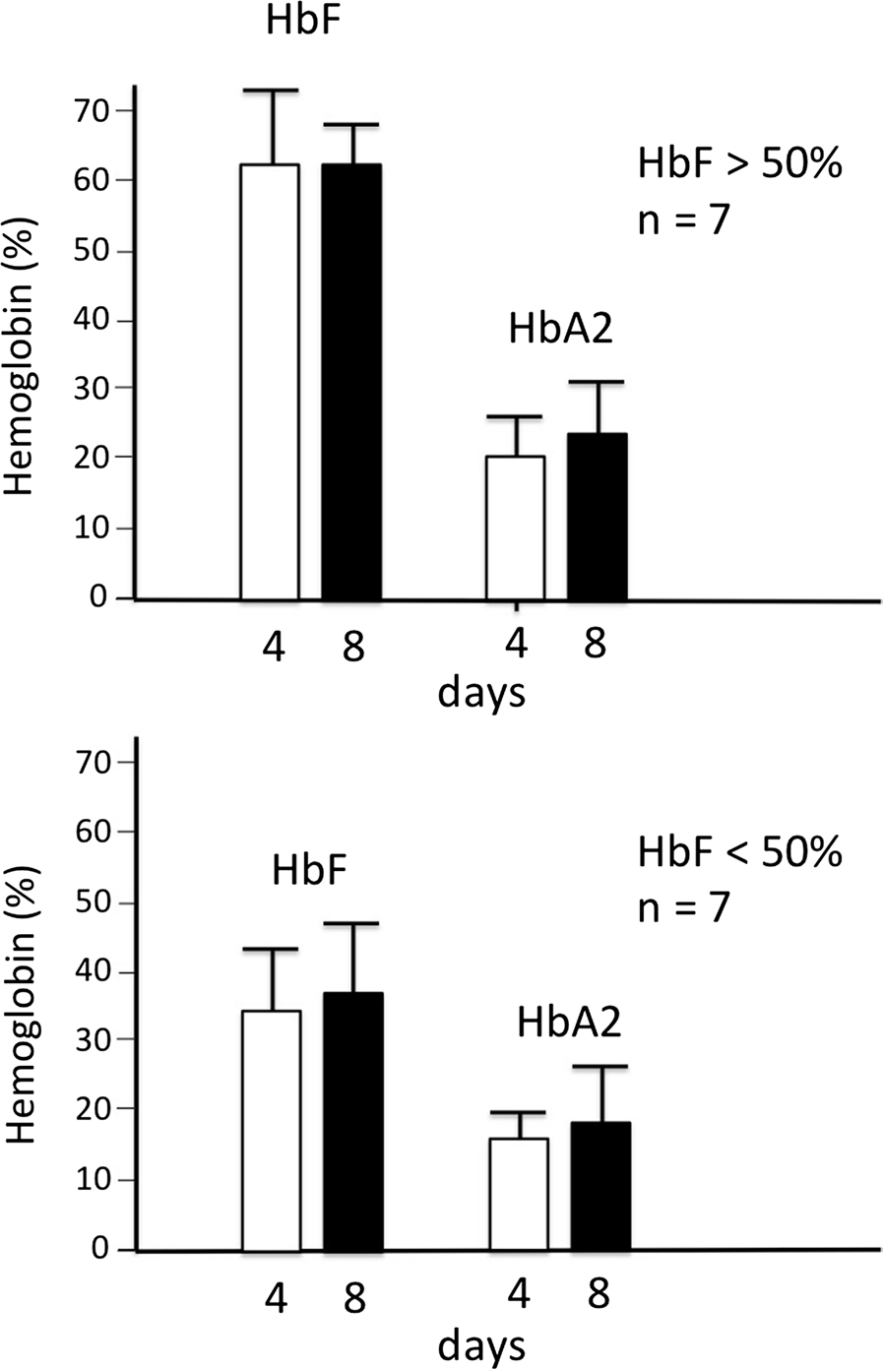
HbF and HbA2 production in ErPC cultures of 14 β-thalassemia and SCA patients after 4 and 8 days differentiation. The hemoglobin levels were analyzed by HPLC and the percentage values obtained indicated. The *upper panel* shows the average ±SD of the samples presenting HbF >50 % and the *lower panel* shows the average of the samples with HbF <50 %, *n* number of ErPC samples

An important validation of the biobanked samples is to show that Hbs types and proportions do not change in cryopreserved samples of the same individual when independently thawed and sub cultured at different time and throughout differentiation. This was confirmed as shown in a representative experiment in Fig. 5f, g. The proportion of HbF and HbA production does not significantly change when vials of same patients are taken from the Thal-Biobank and the cells are thawed and differentiated in vitro. Along with monitoring CD117, CD44, CD29 and GPA antigens expression, we analyzed by HPLC the Hb pattern after 4 and 8 days in cellular cultures from 14 patients exhibiting low or high endogenous levels of HbF production. A summary of the data is reported in Fig. 6 and all the data in Additional file 2: Table S2, which clearly show a good reproducibility in production of the different hemoglobins, with few exceptions (i.e. AVLTF, AVLT23, Fe27 and Fe57, see Additional file 2: Table S2).

### Biobanked samples thawed and subcultured in different laboratories maintain similar patterns of hemoglobin production

**Fig. 7:**
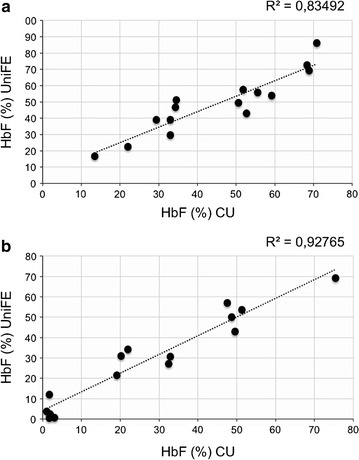
Correlation between cell cultures from the cryopreserved vials from the same subject performed in different research laboratories. HPLC analysis performed on 15 patients (β^0^-thalassemia and β^+^-thalassemia) in two different laboratories, at University of Ferrara (UniFE) and at the Cornell University in New York City (CU). Correlation between the % values of HbF (**a**) and HbA (**b**) evaluated in the cells cultured in the different laboratories

We next determined whether biobanked samples delivered frozen to different laboratories, thawed, subcultured and analyzed for hemoglobin content maintain the same phenotype. The results of this set of experiments are shown in Fig. 7 and formally demonstrate that the hemoglobin pattern is fairly reproduced in different laboratories (in the case reported at the Biotechnology Center of Ferrara University and at the Weill Medical College of Cornell University, NY). A significant correlation was found for both HbF (Fig. 7a) and HbA (Fig. 7b) productions.

### Final validation: induction of fetal hemoglobin (HbF) using biobanked samples

**Fig. 8:**
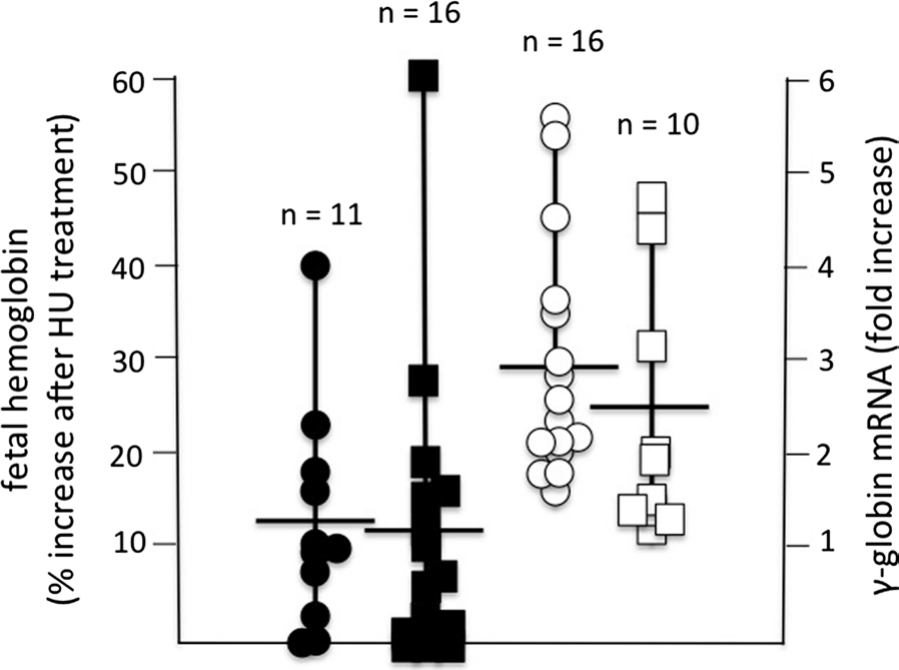
Analysis of HbF production and γ–globin mRNA expression in ErPCs from the cryopreserved vials treated by HU and cultured using both two-phase Fibach’s method (*circles*) and our protocol C (*squares*). In *black*, are reported the % of HbF increase (analyzed by HPLC), in *white*, the fold increase of γ–globin mRNA expression (analyzed by RT-qPCR), *n* number of ErPC samples

To assess the versatility of this system in HbF induction studies, we induced specimens with HU and isolated their RNA content. On these we performed RT-qPCR analysis (in order to quantify the fold induction of γ-globin mRNA) while cytoplasmic extract were used for HPLC analysis (in order to quantify the induction of HbF). The analysis was performed using ErPCs from groups of β^0^- and β^+^-thalassemia patients. The majority of β^0^-thalassemia patients were homozygous β^0^-39/β^0^-39 (29/65), while the majority of the β^+^-thalassemia patients were characterized by a β^+^-IVSI-110 genotype (8/65 homozygous and 20/65 heterozygous, see Fig. 1). In addition we analyzed specimens from 7 patients with compound βS/β thal or homozygote βS/S SCA. The results obtained using protocol C were compared with the results obtained using the two-phase protocol developed by the Fibach’s group. In evaluating HbF variations we used the algorithm: % of HbF increase = (%HbF induced cells − %HbF uninduced cells)/(100 − %HbF uninduced cells) × 100. This algorithm accounts for different proportion of HbF in different samples at steady state in our culture conditions (see Fig. 4). Following this algorithm, induction of HbF was quantified in biobanked samples induced with HU using protocol C. The distribution of HbF induction was similar to that obtained using the two-phases protocol. The results on fold increase of γ-globin mRNA in HU treated cells are shown in Fig. 8, which shows that the induction of γ-globin mRNA obtained with protocol C was, as expected, heterogeneous, yet with a trend comparable to that obtained using the two-phase protocol.

## Discussion

Cellular biobanking is an important approach for collaborative networks planning to use same cells in studies aimed at solving different biological and biomedical issues [2, 23]. This is of key interest in studies on β-thalassemia, since the recruitment of patients and the needed blood drawing may present important limitations in the experimental planning. It should be noted that the commonly used approach is based on (a) recruitment of patients; (b) withdrawal of a 20–25 ml aliquot of blood before transfusion (please note that each patient usually has a personalized schedule for blood transfusion and therefore it is very difficult to treat in parallel erythroid precursor isolated from large numbers of patients); (c) culturing in vitro for a limited length of time; (d) use of cultures for determining the effects of HbF inducers as well as gene therapy approaches.

On the contrary, the use of biobanked samples allows simultaneous comparison of many potentially therapeutic treatments on several samples growing in parallel.

The biobanked samples were validated for retention of the phenotype in relation to hemoglobin production after thawing (a) frozen cells isolated from a same patient at different times (see Fig. 5a–e) and (b) different frozen batches of a same patient’s cells (see Fig. 5f, g). This reproducibility was found in cultures of the same biobanked cells that were sent to different laboratories (see Fig. 7). The use of biobanked cells allows stratification of the patients with respect to HbF production. Finally, the biobanked cells can be used for determining the response to pharmacological or other HbF inducers (Fig. 8). As far as characterization of other CFU-e and BFU-e markers, the data obtained so far are compatible with a BFU-e → CFUe progression during the differentiation phase following thawing of cryopreserved cell and sub-culturing with SCF/EPO, characterized by decrease of CD29, CD44 and CD117 expression and increase of GPA. Further experiments are necessary to verify how the kinetics of these changes are related to other published and validated cellular culture systems [20–22]. This characterization will be mandatory in the case other parameters (in addition to hemoglobin production and response to HbF inducers, which were the major objectives of the present study) and will be considered for patient stratification and response to therapy. Transcriptomics and proteomics data will be also very useful in this context.

The possibility to conduct pre-clinical testing on cells of a patient from the cellular Biobank represents an important resource to study the response to novel HbF inducers. In the future, if our system will be validated also for other erythroid-associated markers, in addition to the already analyzed CD117, CD44, CD29, GPA and CD71, this research activity will allow patients stratification taking into account all the phenotypic/genotypic characteristics of individual ErPCs in association with in vitro HbF induction, under treatment with effective inducers.

Further, the cellular Biobank can provide a cell template for the generation of induced pluripotent cells (iPS) for each patient [24, 25]. This is a great opportunity, when considered together with the possibility of gene editing, which could lead to the correction of the genetic mutation by several approaches, including gene therapy and gene editing [26–31]. While up to now the cellular targets of gene therapy have been human erythropoietic stem cells in most studies, in the future we expect that induced pluripotent stem cells (iPSCs) from β-thalassemia patients will be also a useful cellular target of gene therapy and gene editing approaches [32]. The cryopreserved biobanked cells, therefore, might represents a very important biological source and resource for investigators, to perform exploratory experiments and generate preclinical data in these fields of investigation.

## Conclusion

We demonstrated that freezing, cryopreservation and thawing steps maintain a steady erythroid differentiation potential of the cells in terms of both kinetics and types of hemoglobin produced. The validation of the cellular Thal-Biobank was consolidated by results obtained in other laboratories on different batches of the cryopreserved cells from the same patients. Also, these specimens provide an important opportunity for the research, to stratify patients based on all their phenotypic/genotypic characteristics, to evaluate the different ability of each individual to respond to inducers of HbF synthesis, and to develop novel therapeutic strategies for β-thalassemia.

## Additional files

10.1186/s12967-016-1016-4 List of the subjects (patients and healthy subjects) present in the Thal-Biobank.
10.1186/s12967-016-1016-4 HbF and HbA2 production in ErPC cultures from 14 β-thalassemia patients after 4 and 8 days differentiation.

## Abbreviations

ErPCs: erythroid precursor cells
RT-qPCR: reverse transcription quantitative PCR
HPLC: high performance liquid chromatography
HbF: fetal hemoglobin
ZFNs: zing finger nucleases
TALENs: transcription-activator-like effector nucleases
CRISPR: clustered regularly interspaced short palindromic repeats
PBMCs: peripheral blood mononuclear cells
DPBS: Dulbecco’s phosphate buffered saline
α-MEM: α-minimal essential medium
PEN-STREP: penicillin–streptomycin
FBS: fetal bovine serum
SCF: stem cell factor
IMDM: Iscove’s modified Dulbecco’s medium
DMSO: dimethyl sulfoxide
HbA: adult hemoglobin
dNTPs: deoxynucleoside triphosphate
PCR: polymerase chain reaction
EPO: erythropoietin
HU: hydroxyurea
IPSCs: induced pluripotent stem cells

## References

[1] Viertler C, Zatloukal K (2008). Biobanking and Biomolecular Resources Research Infrastructure (BBMRI). Implications for pathology. Pathologe.

[2] Hewitt RE (2011). Biobanking: the foundation of personalized medicine. Curr Opin Oncol.

[3] Riegman PHMM, Betsou F, de Blasio P, Geary P (2008). Biobanking for better healthcare. Mol Oncol.

[4] Leung EL, Cao ZW, Jiang ZH, Zhou H, Liu L (2013). Network-based drug discovery by integrating systems biology and computational technologies. Brief Bioinform.

[5] Myers CD, Katz FE, Joshi G, Millar JL (1984). A cell line secreting stimulating factors for CFU-GEMM culture. Blood.

[6] Fibach E, Manor D, Oppenheim A, Rachmilewitz EA (1989). Proliferation and maturation of human erythroid progenitors in liquid culture. Blood.

[7] Fibach E (2001). Cell culture and animal models to screen for promising fetal hemoglobin-stimulating compounds. Semin Hematol.

[8] Fibach E (1998). Techniques for studying stimulation of fetal hemoglobin production in human erythroid cultures. Hemoglobin.

[9] Breda L, Kleinert DA, Casu C, Casula L, Cartegni L, Fibach E (2011). A preclinical approach for gene therapy of beta-thalassemia. Ann NY Acad Sci.

[10] Zuccato C, Breda L, Salvatori F, Breveglieri G, Gardenghi S, Bianchi N (2012). A combined approach for beta-thalassemia based on gene therapy-mediated adult hemoglobin (HbA) production and fetal hemoglobin (HbF) induction. Ann Hematol.

[11] Lombardo A, Cesana D, Genovese P, Di Stefano B, Provasi E, Colombo DF (2011). Site-specific integration and tailoring of cassette design for sustainable gene transfer. Nat Methods.

[12] Mussolino C, Morbitzer R, Lutge F, Dannemann N, Lahaye T, Cathomen T (2011). A novel TALE nuclease scaffold enables high genome editing activity in combination with low toxicity. Nucleic Acids Res.

[13] Dong S, Lin J, Held NL, Clem RJ, Passarelli AL, Franz AW (2015). Heritable CRISPR/Cas9-mediated genome editing in the yellow fever mosquito, *Aedes aegypti*. PLoS ONE.

[14] Lin Y, Fine EJ, Zheng Z, Antico CJ, Voit RA, Porteus MH (2014). SAPTA: a new design tool for improving TALE nuclease activity. Nucleic Acids Res.

[15] Gaj T, Gersbach CA, Barbas CF (2013). ZFN, TALEN, and CRISPR/Cas-based methods for genome engineering. Trends Biotechnol.

[16] Bradai M, Abad MT, Pissard S, Lamraoui F, Skopinski L, de Montalembert M (2003). Hydroxyurea can eliminate transfusion requirements in children with severe beta-thalassemia. Blood.

[17] Alebouyeh M, Moussavi F, Haddad-Deylami H, Vossough P (2004). Hydroxyurea in the treatment of major beta-thalassemia and importance of genetic screening. Ann Hematol.

[18] Santoni de Sio F, Naldini L. Short-term culture of human CD34^+^ for lentiviral gene transfer. In: Christopher B, editor. Methods in molecular biology, methods and protocol. New York: Human Press; 2009. doi:10.1.007/978-1-59745-409-4-5. 10.1007/978-1-59745-409-4_5 19110619

[19] Fibach E, Bianchi N, Borgatti M, Zuccato C, Finotti A, Lampronti I (2006). Effects of rapamycin on accumulation of alpha-, beta- and gamma-globin mRNAs in erythroid precursor cells from betathalassaemia patients. Eur J Haematol.

[20] Chen K, Liu J, Heck S, Chasls JA, An X, Mohandas N (2009). Resolving the distinct stages in erythroid differentiation based on dynamic changes in membrane protein expression during erythropoiesis. Proc Natl Acad Sci USA.

[21] Li J, Hale J, Bhagia P, Xue F, Chen L, Jaffray J, Yan H, Lane J, Gallegher PG, Mohandas N, Liu J, An X (2014). Isolation and transcriptome analyses of human erythroid progenitors: BFU-E and CFU-E. Blood.

[22] Mori Y, Chen JY, Pluvinage JV, Seita J, Weissman IL (2015). Prospective isolation of human erythroid lineage-committed progenitors. Proc Natl Acad Sci USA.

[23] Kohane IS (2011). Using electronic health records to drive discovery in disease genomics. Nat Rev Genet.

[24] Takahashi A, Tokunaga A, Yamanaka H, Mashimo T, Noguchi K, Uchida I (2006). Two types of GABAergic miniature inhibitory postsynaptic currents in mouse substantia gelatinosa neurons. Eur J Pharmacol.

[25] Yamanaka S, Takahashi K (2006). Induction of pluripotent stem cells from mouse fibroblast cultures. Tanpakushitsu Kakusan Koso.

[26] Kass EM, Jasin M (2010). Collaboration and competition between DNA double-strand break repair pathways. FEBS Lett.

[27] Breda L, Casu C, Gardenghi S, Bianchi N, Cartegni L, Narla M (2012). Therapeutic hemoglobin levels after gene transfer in beta-thalassemia mice and in hematopoietic cells of beta-thalassemia and sickle cells disease patients. PLoS ONE.

[28] Sadelain M, Boulad F, Lisowki L, Moi P, Riviere I (2008). Stem cell engineering for the treatment of severe hemoglobinopathies. Curr Mol Med.

[29] Sadelain M, Lisowski L, Samakoglu S, Rivella S, May C, Riviere I (2005). Progress toward the genetic treatment of the beta-thalassemias. Ann NY Acad Sci.

[30] Finotti A, Breda L, Lederer CW, Bianchi N, Zuccato C, Kleanthous M (2015). Recent trends in the gene therapy of beta-thalassemia. J Blood Med.

[31] Mussolino C, Cathomen T (2012). TALE nucleases: tailored genome engineering made easy. Curr Opin Biotechnol.

[32] Ma N, Liao B, Zhang H, Wang L, Shan Y, Xue Y (2013). Transcription activator-like effector nuclease (TALEN)-mediated gene correction in integration-free beta-thalassemia induced pluripotent stem cells. J Biol Chem.

