# Supplementary material for: A validated cellular biobank for β-thalassemia
Source: J Transl Med. 2016 Sep 2;14(1):255. doi: 10.1186/s12967-016-1016-4 (PMC5010737; doi:10.1186/s12967-016-1016-4)
Supplement: Supplementary file 1 — 10.1186/s12967-016-1016-4 List of the subjects (patients and healthy subjects) present in the Thal-Biobank. [file 12967_2016_1016_MOESM1_ESM.docx]

**Table S1.** **List of the specimens from thalassemia patients and healthy donors for the Thal-Biobank**. Each subject is associated to a unique code and the β-globin genotype. Polymorphisms in BCL11A (*rs*1427407, *rs*10189857), HS1L-cMYB (*rs*9399137) and XmnI (*rs*7482144) are indicated.

| **CODE** | **GENOTYPE** | **XmnI**  *rs*7482144 | **BCL11A**  *rs*1427407 | **BCL11A**  ***r****s*10189857 | **HS1L-cMYB**  *rs*9399137 |
| --- | --- | --- | --- | --- | --- |
| D1 | βwt/βwt | +/- | GG | AG | TT |
| D2 | βwt/βwt | +/- | GG | GG | TT |
| D3 | βwt/βwt | +/- | GT | AG | TT |
| D4 | βwt/βwt | +/- | GG | AG | TT |
| D5 | βwt/βwt | -/- | GG | GG | TT |
| D6 | βwt/βwt | +/- | GT | AG | TT |
| D7 | βwt/βwt | +/- | GG | AA | CC |
| D8 | βwt/βwt | +/+ | GG | GG | TT |
| Fe2 | β^+^IVSI-110/β^+^IVSI-110 | -/- | GG | GG | TC |
| Fe3 | β^+^IVSI-110/β^0^39 | +/- | GG | AG | TT |
| Fe6 | β^0^39/β^0^39 | -/- | GG | AG | TT |
| Fe8 | β^0^39/β^0^39 | +/- | GT | AG | TT |
| Fe9 | β^0^39/β^0^39 | +/- | GG | GG | TT |
| Fe10 | β^+^IVSI-6/β^0^39 | -/- | GG | AA | TT |
| Fe13 | β^0^39/β^0^39 | -/- | GT | AG | TC |
| Fe15 | β^0^39/β^0^39 | -/- | GT | AG | TC |
| Fe18 | β^0^39/β^0^39 | -/- | GG | AG | TT |
| Fe21 | β^0^39/β^0^39 | -/- | TT | AA | TT |
| Fe23 | β^+^IVSI-110/β^0^IVSI-1 | -/- | GG | AA | TC |
| Fe24 | β^+^IVSI-110/β^+^IVSI-110 | -/- | GG | GG | TT |
| Fe25 | β^+^IVSI-110/β^+^IVSI-110 | -/- | GG | AA | TC |
| Fe27 | β^0^39/β^0^39 | -/- | TT | AA | TT |
| Fe28 | β^0^39/β^0^44 | +/- | GT | AG | TC |
| Fe29 | β^0^39/β^0^39 | +/- | TT | AA | TT |
| Fe32 | β^0^39/β^0^39 | +/- | GG | AG | TT |
| Fe33 | β^+^IVSI-110/ β^0^39 | -/- | GG | AA | TT |
| Fe34 | β^+^IVSI-110/β^0^39 | +/- | GT | AG | TT |
| Fe35 | β^+^IVSI-110/β^+^IVSI-110 | -/- | GG | AA | TC |
| Fe37 | β^+^IVSI-110/β^+^IVSI-110 | +/- | GG | AG | TT |
| Fe38 | β^+^IVSI-6/β^+^IVSI-6 | -/- | GG | AA | TC |
| Fe42 | β^+^IVSI-110/β^0^39 | -/- | GG | GG | TT |
| Fe43 | β^+^IVSI-6/β^+^IVSI-110 | -/- | GT | AG | TC |
| Fe44 | β^0^39/β^0^39 | +/+ | GT | GG | TT |
| Fe46 | β^0^39/β^0^39 | -/- | GT | AG | TT |
| Fe50 | β^0^39/β^0^39 | -/- | GG | AG | TT |
| Fe52 | β^+^IVSI-110/β^+^IVSI-110 | -/- | GG | AG | TC |
| Fe57 | β^+^IVSI-6/β^+^IVSI-6 | -/- | GG | AA | TT |
| Fe64 | HbS/β^0^39 | -/- | GG | AA | TC |
| Fe67 | HbS/β^+^IVSI-6 | -/- | GG | AG | TT |
| Fe69 | HbS/HbS | -/- | GG | AG | TT |
| Fe73 | β^0^39/β^0^39 | +/- | GG | GG | TC |
| Fe75 | HbS/β^+^IVSI-110 | -/- | GG | AG | TT |
| Fe76 | β^0^39/β^0^39 | -/- | GT | AA | TC |
| Fe77 | β^0^39/β^0^39 | +/+ | GG | AA | CC |
| Fe78 | HbS/β^0^IVSI-1 | -/- | GG | AA | TC |
| Fe80 | β^+^IVSI-110/β^0^39 | -/- | GG | AG | TC |
| Fe81 | HbS/HbS | -/- | GG | AA | TT |
| Fe82 | HbS/β^0^IVSI-1 | -/- | GG | AG | TC |
| AVLTA | β^+^IVSI-6/β^0^39 | -/- | GG | AA | TT |
| AVLTC | β^0^39/β^+^5’UTR+20nt | -/- | GT | AG | TT |
| AVLTD | β^+^IVSI-110/β^0^39 | -/- | GG | AG | TT |
| AVLTE | β^+^IVSI-110/β^0^39 | -/- | GT | AA | TC |
| AVLTF | β^0^39/β^0^39 | +/- | GT | AA | TC |
| AVLTG | β^+^IVSI-110/β^0^39 | -/- | GG | AG | TC |
| AVLTH | β^0^39/β^0^39 | -/- | GG | AG | TT |
| AVLTL | β^+^IVSI-6/β^0^39 | -/- | GG | AA | TT |
| AVLTM | β^0^39/β^0^39 | +/+ | GG | AG | TT |
| AVLTN | β^+^IVSI-110/β^0^39 | -/- | GT | AA | TC |
| AVLTO | β^0^39/β^0^28(+C) | -/- | GT | AA | TT |
| AVLTP | β^0^39/β^0^39 | -/- | GT | AG | TC |
| AVLTQ | β^+^IVSI-6/β^0^39 | -/- | GG | AG | TT |
| AVLTR | β^0^39/β^0^39 | -/- | GG | AA | TC |
| AVLTS | β^0^39/β^0^39 | -/- | GG | AG | TC |
| AVLTT | β^0^39/β^0^39 | -/- | GG | AG | TC |
| AVLTU | β^0^39/β^0^39 | -/- | GG | AA | TT |
| AVLTV | β^+^IVSI-110/β^+^IVSI-110 | -/- | GG | AG | TC |
| AVLT21 | β^0^39/β^0^39 | -/- | GG | AA | CC |
| AVLT22 | β^+^IVSI-110/β^0^39 | -/- | GG | AG | TT |
| AVLT23 | β^+^IVSI-110/β^0^39 | -/- | GG | AG | CC |
| AVLT25 | β^0^39/β^0^39 | -/- | GG | AA | TT |
| AVLT28 | β^+^IVSI-110/β^0^39 | -/- | GG | AG | TT |
| AVLT29 | β^+^IVSI-110/β^0^39 | -/- | GT | AA | TT |
| AVLT31 | β^+^IVSI-110/β^0^39 | -/- | GT | AG | TC |
| AVLT32 | β^0^39/β^0^39 | +/- | GG | AG | TT |
| AVLT33 | β^0^39/β^0^39 | -/- | GT | AA | TC |
| AVLT34 | β^+^IVSI-110/β^0^39 | -/- | GG | AG | TC |
| AVLT35 | β^+^IVSI-110/β^0^39 | -/- | GG | GG | TC |
| AVLT36 | β^+^IVSI-110/β^0^39 | -/- | GG | AG | TT |
| AVLT37 | β^0^39/β^0^39 | -/- | GT | AA | TT |
| AVLT39 | β^+^IVSI-110/β^+^IVSI-110 | -/- | GG | AG | TT |
