# Supplementary material for: A validated cellular biobank for β-thalassemia
Source: J Transl Med. 2016 Sep 2;14(1):255. doi: 10.1186/s12967-016-1016-4 (PMC5010737; doi:10.1186/s12967-016-1016-4)
Supplement: Supplementary file 2 — 10.1186/s12967-016-1016-4 HbF and HbA2 production in ErPC cultures from 14 β-thalassemia patients after 4 and 8 days differentiation. [file 12967_2016_1016_MOESM2_ESM.docx]

**Table S2.** **HbF and HbA2 production in ErPC cultures from 14 β-thalassemia patients after 4 and 8 days differentiation**. Each ErPC culture was analysed for hemoglobin production by HPLC after 4 and 8 days of differentiation post-cryopreservation and thawing. The ErPCs cultures were grouped depending from their HbF endogenous levels above and below 50%

| **A: ErPCs producing high endogenous levels of HbF >50%** | | | | | | | |
| --- | --- | --- | --- | --- | --- | --- | --- |
| **CODE** | **GENOTYPE** | %HbF | | | %HbA2 | | |
|  |  | day 4 | day 8 | variation (%) | day 4 | day 8 | variation (%) |
| AVTLF | β^0^39/β^0^39 | 64,83 | 54,41 | -10,42 | 30,5 | 26,46 | -4,04 |
| Fe29 | β^0^39/β^0^39 | 60,44 | 63,28 | 2,84 | 22,88 | 24,73 | 0,15 |
| Fe6 | β^0^39/β^0^39 | 59,71 | 67,4 | 7,69 | 26,98 | 21,52 | -5,46 |
| Fe46 | β^0^39/β^0^39 | 57,78 | 63,17 | 5,39 | 26,17 | 21,04 | -5,13 |
| AVLT23 | β^0^39/β^+^IVSI-110 | 59,28 | 48,19 | -11,09 | 22,78 | 38,77 | 15,99 |
| AVLT24 | β^0^39/β^0^39 | 61,88 | 63,28 | 1,4 | 14,06 | 24,76 | 10,7 |
| Fe62 | β^+^IVSI-110/βwt | 65,76 | 69,92 | 4,16 | 5,44 | 2,47 | -2,97 |
|  | | | | | | | |
| **B: ErPCs producing endogenous levels of HbF <50%** | | | | | | | |
|  |  | %HbF | | | %HbA2 | | |
| **CODE** | **GENOTYPE** | day 4 | day 8 | variation (%) | day 4 | day 8 | variation (%) |
| Fe78 | β^0^IVSI-1/HbS | 46,64 | 51,75 | 5,11 | 13,50 | 8,42 | -5,08 |
| AVLTR | β^0^39/β^0^39 | 29,2 | 42,88 | 13,68 | 29,12 | 34,69 | 5,57 |
| Fe27 | β^0^39/β^0^39 | 41,16 | 37,9 | -3,26 | 26,09 | 42,50 | 16,41 |
| AVLTA | β^0^39/β^+^IVSI-6 | 32,99 | 34,95 | 1,96 | 13,12 | 15,99 | 2,87 |
| Fe37 | β^+^IVSI-110/ β^+^IVSI-110 | 47,56 | 57,52 | 9,96 | 14,28 | 9,01 | -5,27 |
| Fe57 | β^+^IVSI-6/β^+^IVSI-6 | 20,7 | 17,4 | -3,3 | 11,35 | 9,65 | -1,7 |
| AVLTQ | β^0^39/β^+^IVSI-6 | 13,64 | 21,44 | 7,8 | 21,14 | 15,72 | -5,42 |
